# Advances in Managing Pelvic Fractures in Polytrauma: A Comprehensive Review

**DOI:** 10.3390/jcm14051492

**Published:** 2025-02-23

**Authors:** Uros Dabetic, Jovana Grupkovic, Slavisa Zagorac, Dejan Aleksandric, Nikola Bogosavljevic, Goran Tulic

**Affiliations:** 1 Clinic for Orthopedic Surgery and Traumatology, University Clinical Center of Serbia, 11000 Belgrade, Serbia; urosdabetic1983@gmail.com (U.D.); slavisa.zagorac@gmail.com (S.Z.); tulic05@gmail.com (G.T.); 2 Faculty of Medicine, University of Belgrade, 11000 Belgrade, Serbia; boga19@gmail.com; 3 Institute for Orthopedic Surgery “Banjica”, 11000 Belgrade, Serbia; aleksandricdejan@gmail.com

**Keywords:** pelvic fractures, polytrauma, damage control orthopedics, early total care, complications, surgical timing, advanced technologies

## Abstract

**Background:** Pelvic fractures are among the most complex and life-threatening injuries encountered in trauma and orthopedic surgery, often resulting from high-energy trauma and leading to severe complications. This review synthesizes recent advancements in pelvic trauma care, with a focus on comparing damage control orthopedics (DCO) and early total care (ETC) strategies, operative versus nonoperative management, and outcomes of minimally invasive versus traditional ORIF techniques. **Results:** Our comparative analysis highlights that DCO remains the preferred approach for hemodynamically unstable patients, prioritizing rapid stabilization and reducing mortality from hemorrhage. In contrast, ETC has demonstrated superior functional recovery outcomes in stable polytrauma patients, with a 30–40% reduction in pulmonary complications and shorter ICU stays when performed within 24–48 h post-injury. Additionally, percutaneous fixation reduces soft tissue trauma and infection risk but increases the likelihood of malunion, while ORIF provides superior anatomical restoration with a higher risk of postoperative infections. Hybrid approaches, integrating percutaneous techniques with limited open reduction, show promise in minimizing operative time and complications while achieving stable fixation. **Conclusions:** These findings reinforce the importance of tailoring surgical strategies to patient physiology and injury patterns. DCO and ETC have distinct but complementary roles, and emerging hybrid techniques offer a middle ground that balances stability with reduced morbidity. A precision medicine approach, integrating AI-driven predictive modeling and real-world clinical data, is essential for optimizing outcomes and developing evidence-based treatment protocols. Large-scale, multicenter trials are needed to validate these approaches and establish standardized guidelines for pelvic fracture management.

## 1. Introduction

Pelvic fractures represent one of the most complex and life-threatening injuries encountered in trauma and orthopedic surgery. These injuries are often the result of high-energy trauma, such as motor vehicle accidents, falls from significant heights, or crush injuries, and they account for a substantial proportion of trauma-related morbidity and mortality worldwide [1]. Given the critical anatomical structures housed within the pelvic ring—including major neurovascular bundles, the urogenital system, and weight-bearing articulations—fractures in this region can lead to severe hemorrhage, multi-organ dysfunction, long-term disability, and death [2].

A central controversy in pelvic fracture management lies in the ongoing debate between damage control orthopedics (DCO) and early total care (ETC), two distinct strategies with significant implications for survival, recovery, and long-term outcomes.

A key point of contention is whether damage control orthopedics (DCO) or early total care (ETC) provides superior outcomes in polytraumatized patients [2,3,4]. While DCO prioritizes rapid stabilization and physiological recovery, ETC advocates for early definitive fixation, aiming to reduce complications such as systemic inflammatory response syndrome (SIRS) and multiple-organ dysfunction syndrome (MODS) [5]. Additionally, the choice between operative and nonoperative management, particularly in borderline unstable fractures, remains controversial, as long-term functional outcomes and complication rates continue to be debated [6].

Another pressing question in pelvic trauma management is the selection of the optimal surgical technique. Percutaneous fixation techniques have gained popularity due to their minimally invasive nature, reduced surgical morbidity, and shorter hospital stays, but concerns persist regarding their biomechanical stability and long-term durability [2,7]. Conversely, open reduction and internal fixation (ORIF) remains the gold standard for achieving anatomical restoration and stable fixation but at the cost of increased surgical trauma and infection risk. Emerging hybrid approaches, which integrate elements of both techniques, are being explored as potential solutions to these limitations.

Given the wide variation in injury patterns, patient physiology, and institutional capabilities, there is no universal consensus on the ideal treatment algorithm for pelvic fractures. This review aims to synthesize current knowledge, analyze recent evidence on surgical timing and techniques, and provide a comparative assessment of operative vs. nonoperative management. By addressing key controversies and research gaps, this review contributes to refining evidence-based treatment protocols and shaping future directions in pelvic trauma care.

## 2. Methodology

A comprehensive literature search was conducted to gather current evidence on the management of pelvic fractures in polytrauma patients. We searched major electronic databases including PubMed, Embase, Scopus, and Web of Science for relevant articles published between January 2009 and December 2024. The search employed a combination of keywords and Medical Subject Headings (MeSH) terms such as “pelvic fractures”, “polytrauma”, “damage control orthopedics (DCO)”, “early total care (ETC)”, “operative management”, “nonoperative management”, “percutaneous fixation”, “open reduction and internal fixation (ORIF)”, and “complications”. In addition, the reference lists of selected articles were reviewed to identify further relevant studies.

### 2.1. Inclusion and Exclusion Criteria

Studies were included if they met the following criteria:

- Peer-reviewed articles published in English.
- Original research articles, systematic reviews, narrative reviews, meta-analyses, and clinical guidelines addressing pelvic fracture management in the context of polytrauma.
- Articles discussing surgical timing, fixation techniques, complications, or outcomes relevant to the topic.

Articles were excluded if they met the following criteria:

- Focusing exclusively on pediatric populations.
- Addressing isolated acetabular fractures without pelvic ring involvement.
- Lacking sufficient methodological detail.

### 2.2. Data Extraction and Synthesis

Two independent reviewers screened titles and abstracts for relevance. Discrepancies were resolved through discussion and consensus. Data were extracted regarding study design, patient demographics, interventions, and outcomes. Due to the heterogeneity of the study designs and reported outcomes, a formal quality assessment and quantitative meta-analysis were not performed. Instead, the findings were synthesized narratively to highlight common themes, areas of controversy, and emerging trends in pelvic fracture management.

By detailing this methodology, we aim to provide clarity and reproducibility in our review process, ensuring that the evidence presented is robust and comprehensive.

## 3. Timing of Surgical Management

The timing of surgical intervention in patients with pelvic fractures is a critical determinant of outcomes, influencing both immediate survival and long-term functionality. Deciding when to proceed with definitive surgical fixation involves a nuanced assessment of the patient’s hemodynamic stability, the severity of associated injuries, and the potential risks of early versus delayed intervention. Two primary strategies have emerged in this context: damage control orthopedics (DCO) and early total care (ETC) [3,4].

### 3.1. Damage Control Orthopedics (DCO)

DCO is a staged approach designed for hemodynamically unstable patients or those with severe polytrauma. The initial phase focuses on rapid stabilization to prevent the “lethal triad” of hypothermia, acidosis, and coagulopathy. This involves the application of external fixation devices to stabilize pelvic fractures, thereby controlling hemorrhage and providing provisional stability [8]. Recent advancements in external fixation technology have led to the development of biomechanically optimized frames that offer improved stability and ease of application. Adjunctive measures, such as pre-peritoneal packing and angiographic embolization, have demonstrated high success rates in controlling pelvic hemorrhage, with some studies reporting efficacy rates exceeding 90% [9,10].

To enhance decision-making during the acute phase, near-infrared spectroscopy has been utilized to assess tissue perfusion, providing real-time feedback on the effectiveness of resuscitative efforts. Advanced hemodynamic monitoring techniques, including continuous cardiac output analysis, guide fluid resuscitation and the administration of blood products, ensure optimized patient outcomes [8,10,11]. Furthermore, the integration of biochemical markers and imaging findings into treatment algorithms offers clinicians actionable insights, facilitating timely interventions.

Recent advancements in artificial intelligence (AI) have led to the development of predictive models that significantly enhance clinical decision-making in pelvic trauma care. For instance, several machine learning algorithms now integrate clinical data—such as vital signs, laboratory values, and CT imaging features—to predict outcomes like hemorrhage risk and the need for massive transfusion. In one multicenter study, a deep learning model demonstrated an area under the curve (AUC) of 0.85 in predicting the need for a massive transfusion in pelvic fracture patients [12]. Furthermore, AI-driven decision support systems have been deployed to assist surgeons in choosing the optimal fixation technique by analyzing patient-specific variables and fracture patterns. Virtual simulation models, which have been clinically validated to improve preoperative planning accuracy, have also shown promise in reducing operative times and enhancing surgical outcomes [13]. These examples underscore the growing role of AI in delivering personalized, data-driven care in the management of pelvic fractures.

### 3.2. Early Total Care (ETC)

ETC advocates for early definitive surgical fixation, typically within 24 to 48 h, in patients who are hemodynamically stable or have been adequately resuscitated. The goal is to achieve anatomical reductions in and the stable fixation of fractures, which can reduce complications associated with prolonged immobilization, such as pulmonary issues and thromboembolic events [4,8]. Minimally invasive surgical techniques, including percutaneous fixation and robotic-assisted procedures, have been developed to minimize soft tissue disruption, thereby reducing postoperative morbidity and facilitating faster recovery [7,10].

Comparative studies have indicated that appropriately timed ETC can decrease intensive care unit dependency and lower the incidence of pulmonary complications and thromboembolic events by 30–40% [4]. Enhanced recovery protocols that integrate multimodal analgesia, thromboprophylaxis, and early nutrition are now routinely incorporated into ETC strategies, further improving functional recovery trajectories.

### 3.3. Hybrid Surgical Approaches

Emerging evidence also suggests that hybrid approaches, combining elements of DCO and ETC, may offer superior outcomes in specific clinical scenarios, particularly in patients who initially present with instability but respond well to resuscitation efforts [14,15].

While conventional percutaneous fixation and open reduction and internal fixation (ORIF) represent two ends of the surgical spectrum, hybrid approaches have emerged to combine the benefits of both techniques. One such approach is percutaneous-assisted ORIF, which involves the initial stabilization of fracture fragments using percutaneous screw fixation followed by limited open exposure to achieve a precise reduction in key fragments. This method minimizes soft tissue dissection while allowing the direct visualization of critical areas, resulting in shorter operative times and reduced blood loss compared to conventional ORIF, thus providing a good clinical outcome [16].

Additionally, the use of advanced intraoperative imaging, such as 3D fluoroscopy and navigation-assisted surgery, further refines these hybrid techniques by ensuring optimal screw placement and alignment, thereby reducing the risk of neurovascular injury [14,17].

Collectively, these hybrid approaches offer a promising middle ground for managing complex pelvic fractures in polytrauma patients, balancing the need for robust mechanical stability with the imperative to minimize surgical morbidity.

### 3.4. Determinants of Surgical Timing

Determining the optimal timing for surgical intervention requires a comprehensive assessment of various clinical parameters. Biomarkers such as lactate clearance and base deficit are valuable indicators of tissue perfusion and the adequacy of resuscitation. Dynamic imaging modalities, including contrast-enhanced computed tomography (CT), provide detailed insights into injury patterns and ongoing hemorrhage, aiding in surgical planning. Advanced imaging techniques, such as four-dimensional (4D) vascular assessments, have augmented diagnostic accuracy by offering a clearer understanding of dynamic bleeding patterns, thereby guiding timely interventions [13].

Anesthetic considerations play a vital role in surgical outcomes, particularly in elderly patients. Recent evidence has demonstrated a significant association between spinal anesthesia and hypotensive events during hip fracture repair, emphasizing the importance of thorough perioperative management in reducing surgical morbidity [18].

The advent of artificial intelligence-driven predictive models has further enhanced decision-making by offering real-time risk stratification. These models analyze a multitude of variables to predict patient trajectories, enabling more individualized and precise management decisions [17]. Additionally, virtual simulation models have been employed in preoperative planning, allowing for the comprehensive evaluation of complex pelvic injuries and facilitating the rehearsal of surgical approaches [19].

In conclusion, the timing of surgical management in pelvic fractures necessitates a tailored approach that considers the patient’s physiological status, injury severity, and response to initial resuscitation. Both DCO and ETC have distinct roles, and the integration of advanced monitoring techniques, minimally invasive procedures, and predictive modeling can aid in optimizing outcomes. Ongoing research and technological advancements continue to refine these strategies, contributing to improved care for patients with pelvic fractures.

The inclusion of virtual simulation models has further enhanced preoperative planning by allowing for the comprehensive evaluation of complex pelvic injuries.

## 4. Operative vs. Nonoperative Treatment

In polytrauma patients, pelvic fractures present a significant challenge, necessitating a nuanced approach to management that balances the benefits and risks of operative versus nonoperative treatments. Recent studies have provided insights into the outcomes associated with each strategy, particularly in specific patient populations.

A retrospective study focusing on stress-positive, minimally displaced, lateral compression type 1 (LC1b) pelvic ring injuries found that operative treatment was associated with early benefits over nonoperative management. These benefits included shorter durations of assistive device use, reduced reliance on subacute rehabilitation facilities, and less fracture displacement at follow-up. However, the study also noted a higher complication rate in the operative group, with 29.6% experiencing complications compared to 25.0% in the nonoperative group. This underscores the importance of careful patient selection when considering surgical intervention [20]. 

Conversely, a study examining young patients with type B2.1 pelvic fractures found no significant difference in short-term functional outcomes or quality of life between operative and nonoperative treatments. Both groups exhibited similar reductions in function and quality of life measures post-injury, indicating that nonoperative management may be appropriate in select cases [21].

These findings highlight the complexity of managing pelvic fractures in polytrauma patients. The decision between operative and nonoperative treatment should be individualized, taking into account factors such as fracture type, patient age, comorbidities, and overall functional status. Further high-quality research is essential to establish clear guidelines and optimize outcomes for this diverse patient population.

## 5. Percutaneous Fixation vs. ORIF in Polytrauma Patients

In the management of pelvic fractures among polytrauma patients, the choice between percutaneous fixation and open reduction and internal fixation (ORIF) is pivotal, with each approach offering distinct advantages and challenges. Percutaneous fixation, characterized by its minimally invasive nature, aims to stabilize fractures with reduced soft tissue disruption, potentially leading to decreased operative times and minimized blood loss. Conversely, ORIF involves the direct visualization and manipulation of fracture fragments, facilitating precise anatomical restoration but often at the cost of increased surgical exposure and associated morbidities.

A study by Tosounidis et al. highlighted the efficacy of external fixation combined with percutaneous screw fixation in polytrauma patients with unstable pelvic ring injuries. This approach provided adequate stabilization while minimizing surgical invasiveness, which is particularly beneficial in patients with compromised physiological reserves. The authors reported satisfactory clinical outcomes with low complication rates, suggesting that minimally invasive techniques can be a viable alternative to traditional open methods in select scenarios [22]. 

In contrast, a comparative study by Einhorn et al. evaluated closed reduction and percutaneous internal fixation (CRPIF) against ORIF in the treatment of acetabular fractures. The findings indicated that CRPIF resulted in less intraoperative blood loss and shorter surgical durations compared to ORIF. However, ORIF demonstrated superior fracture reduction, which is a critical factor in joint congruency and long-term function. Despite these differences, both groups exhibited comparable rates of conversion to total hip arthroplasty and similar functional outcomes at a one-year follow-up, underscoring the importance of individualized treatment planning based on patient-specific factors and fracture characteristics [7]. 

Further supporting the benefits of minimally invasive approaches, a study by Lee et al. reported on the use of percutaneous screw fixation and external stabilization as definitive treatment in a severely injured polytrauma patient with an unstable pelvic ring injury. The patient achieved successful fracture healing and functional recovery without the need for subsequent open procedures, highlighting the potential of percutaneous methods in managing complex pelvic injuries in critically ill patients [23]. 

Collectively, these studies suggest that while ORIF remains the gold standard for achieving optimal anatomical reduction in pelvic fractures, percutaneous fixation techniques offer a less invasive alternative with reduced perioperative morbidity. The decision between percutaneous fixation and ORIF should be guided by factors such as fracture type, patient stability, comorbid conditions, and the surgeon’s expertise. In polytrauma patients, where minimizing surgical burden is often paramount, percutaneous methods may provide sufficient stabilization with a favorable risk profile. Nonetheless, meticulous patient selection and adherence to surgical principles are essential to ensure successful outcomes.

## 6. Complications of Pelvic Fractures

Pelvic ring fractures, due to their complex anatomy and high-energy mechanisms, are associated with a range of complications that significantly impact patient outcomes. These complications can be broadly categorized into early and late manifestations, each with distinct clinical implications.

### 6.1. Early Complications

#### 6.1.1. Hemorrhage

Hemorrhage remains the most urgent complication, occurring from the venous plexus or arterial sources. The venous plexus in the posterior pelvis accounts for approximately 90% of hemorrhage associated with pelvic ring injuries, with arterial injuries, though less common, contributing significantly to morbidity. While hemorrhage control strategies such as external fixation, pre-peritoneal packing, and angiographic embolization are discussed in the DCO section, it is crucial to note that rapid mechanical stabilization significantly reduces mortality. Severe retroperitoneal bleeding can lead to hemodynamic collapse, underscoring the importance of immediate intervention [21].

#### 6.1.2. Thromboembolic Events

Deep vein thrombosis (DVT) and pulmonary embolism (PE) are prevalent due to immobility and venous stasis, with DVT rates reported up to 60% [22]. Prophylactic measures, including mechanical compression and low-molecular-weight heparin, are vital. As discussed under ETC, early mobilization can significantly reduce thromboembolic risk.

#### 6.1.3. Infection

Postoperative infections, particularly following open reduction and internal fixation (ORIF), are a significant concern. Risk factors contributing to postoperative infections include prolonged operative times, extended intensive care unit stays, and the necessity for multiple blood transfusions [23]. Minimally invasive techniques, as outlined in the percutaneous fixation section, may reduce infection rates by limiting soft tissue disruption.

### 6.2. Late Complications

#### 6.2.1. Malunion or Nonunion

The malunion or nonunion of pelvic fractures can lead to chronic pain, gait abnormalities, and functional impairments. The development of post-traumatic arthritis is a recognized sequela, particularly in cases where the articular surfaces are involved or anatomical alignment is not adequately restored. Surgical intervention aimed at achieving precise reduction and stable fixation is paramount in preventing these outcomes [24]. 

#### 6.2.2. Neurological Complications

Arising from injury to the lumbosacral plexus or sacral nerve roots, neurological complications can result in persistent pain, sensory deficits, or motor dysfunction. The L5 and S1 nerve roots are particularly vulnerable due to their anatomical course over the sacral ala and near the sacroiliac joint. Comprehensive neurological assessment and, when indicated, early neurosurgical consultation are essential components of care [25,26]. 

#### 6.2.3. Urogenital Injuries

These are reported in approximately 12–20% of patients with pelvic fractures, with a higher incidence observed in males. These injuries can include posterior urethral tears and bladder ruptures, necessitating prompt diagnosis through appropriate imaging modalities and timely surgical repair to prevent long-term sequelae such as stricture formation or incontinence [27].

#### 6.2.4. Sexual Dysfunction

This is a recognized complication following pelvic fractures, with varying incidence rates reported in the literature. A systematic review indicated that the severity and frequency of sexual dysfunction are associated with the type of pelvic ring fracture, particularly in cases involving higher energy trauma. The study emphasized the importance of electrophysiological assessments in patients experiencing sexual dysfunction post-injury [28].

The management of sexual dysfunction in these patients necessitates a multidisciplinary approach. Interventions may include reconstructive surgery to address anatomical injuries and psychosexual therapy to support psychological well-being. Early identification and comprehensive treatment strategies are essential to improve sexual health outcomes in this population.

#### 6.2.5. Mobility Impairment

This is another significant late complication of pelvic fractures. Patients may experience persistent pain, gait abnormalities, and reduced physical activity levels, leading to long-term disability. A randomized controlled trial evaluated the impact of an exercise self-management intervention on mobility-related disability and falls after lower limb or pelvic fractures. While the primary outcomes did not show statistically significant improvements, secondary measures indicated benefits in balance, mobility, physical activity, and health status [29].

These findings suggest that tailored exercise programs focusing on weight-bearing balance and strength training can be beneficial. Implementing such interventions as part of a comprehensive rehabilitation plan may enhance functional recovery and reduce the risk of long-term mobility impairments in patients recovering from pelvic fractures.

In conclusion, the management of pelvic ring fractures requires a comprehensive understanding of the potential complications inherent to these injuries. The early identification and proactive management of both early and late complications are critical to optimizing patient outcomes and minimizing long-term morbidity.

## 7. Discussion

The management of pelvic fractures in polytrauma patients necessitates a multidimensional approach, integrating optimal surgical timing, fixation techniques, and rehabilitation strategies. This review synthesizes key findings, yet it is imperative to place these elements in a comparative framework to underscore their clinical significance. By evaluating the interplay between damage control orthopedics (DCO) and early total care (ETC), operative versus nonoperative strategies, and percutaneous fixation versus ORIF, we can refine treatment algorithms to optimize patient outcomes.

### 7.1. Timing of Surgical Intervention: DCO vs. ETC

The debate between DCO and ETC highlights the inherent tension between immediate stabilization and early definitive care. While DCO remains indispensable in hemodynamically unstable patients, its staged approach often delays definitive fixation, increasing the risk of prolonged immobilization and its associated complications. In contrast, ETC reduces the burden of secondary surgeries and accelerates functional recovery but may pose risks in borderline stable patients [3,4,5,8,14]. The emergence of hybrid models, which incorporate elements of both strategies, suggests that rigid adherence to a single protocol may be suboptimal [22,23]. Future research should focus on refining patient selection criteria to personalize treatment strategies based on real-time physiological indicators.

### 7.2. Operative vs. Nonoperative Management: Rethinking Selection Criteria

Although nonoperative management remains viable for stable pelvic fractures, its applicability in polytrauma patients is limited. The decision to operate must weigh the risks of surgical morbidity against the complications of prolonged nonoperative care, such as thromboembolism and chronic instability. While some studies report comparable functional outcomes between operative and nonoperative groups, these findings are largely confined to young, hemodynamically stable patients. For unstable fractures, early surgical intervention remains paramount [20,21]. The ongoing challenge lies in integrating predictive tools—such as AI-based risk stratification—to refine selection criteria, ensuring that surgical intervention is neither over- nor underutilized.

### 7.3. Percutaneous Fixation vs. ORIF: A Trade-Off Between Stability and Invasiveness

Minimally invasive techniques, including percutaneous fixation, have gained traction due to their reduced perioperative morbidity. However, concerns remain regarding their biomechanical stability, particularly in high-energy fractures. ORIF, by contrast, offers superior anatomical reduction but at the cost of increased soft tissue disruption and infection risk [6,7]. The decision-making process must balance these factors, particularly in polytrauma patients, where minimizing surgical burden is critical. The evolution of hybrid techniques—such as percutaneous-assisted ORIF—may offer a middle ground, optimizing stability while preserving soft tissue integrity.

### 7.4. Complications: Weighing Risks Across Treatment Modalities

The complications associated with pelvic fractures vary significantly based on treatment modality [26]. Early complications such as hemorrhage and thromboembolism are prevalent in both operative and nonoperative groups, though the latter carries an increased risk due to prolonged immobilization [23,24]. Late complications, including malunion, post-traumatic arthritis, and neurological deficits, highlight the importance of precise anatomical reduction [27,28,29]. Sexual dysfunction and urogenital injuries further reinforce the need for comprehensive rehabilitation programs tailored to address both functional and psychosocial dimensions of recovery [30,31,32].

Percutaneous fixation is associated with reduced soft tissue trauma and shorter hospital stays; however, it carries notable risks, including screw malposition, neurovascular injury, and a higher incidence of malunion due to limited fracture visualization. Booth et al. support the use of percutaneous fixation with closed reduction, noting decreased infection rates and shorter lengths of stay. In contrast, open reduction and internal fixation (ORIF) provides superior anatomical restoration but is linked to higher infection rates and increased surgical morbidity [33]. A study by Kanakaris et al. identified key risk factors for deep infections following ORIF, including high overall injury severity, diabetes, alcohol consumption, and the use of posterior approaches for pelvic ring fixation [34]. When comparing outcomes, percutaneous fixation offers a lower risk of infection but a higher risk of neurological injury. As such, the choice between these techniques requires careful consideration of patient-specific factors, fracture patterns, and the surgeon’s experience to ensure an optimal balance between safety and efficacy.

### 7.5. Future Directions: Toward an Integrated, Data-Driven Approach

Looking ahead, pelvic trauma care must embrace a precision medicine framework that integrates real-time data analytics, AI-driven decision support, and robotic-assisted surgical techniques. The incorporation of machine learning models in preoperative planning, intraoperative navigation systems, and postoperative rehabilitation may bridge existing gaps in care [13,17,19]. Additionally, large-scale, multicenter trials are essential to validate emerging hybrid treatment strategies and establish standardized guidelines that balance stability, functional outcomes, and surgical burden.

### 7.6. Limitations

While this review provides a comprehensive synthesis of pelvic fracture management strategies, certain limitations must be acknowledged. First, the heterogeneity of study designs and patient populations included in the reviewed literature limits the ability to generalize findings across all clinical scenarios. Additionally, the absence of large-scale randomized controlled trials comparing hybrid surgical approaches constrains definitive conclusions regarding optimal fixation techniques. Finally, data on the long-term outcomes of minimally invasive techniques remain limited, necessitating future research to validate their efficacy over traditional ORIF.

## 8. Conclusions

This review highlights the critical insights gained from comparing surgical techniques, timing strategies, and hybrid approaches in managing pelvic fractures. The findings emphasize that percutaneous fixation offers reduced soft tissue trauma and shorter recovery times, while ORIF ensures superior anatomical restoration, guiding clinicians in selecting techniques based on patient-specific factors. Additionally, the discussion on DCO versus ETC reinforces the importance of tailoring surgical timing to hemodynamic status, balancing rapid stabilization with early definitive care. Insights into hybrid approaches reveal their potential to minimize complications and improve functional recovery by integrating the strengths of percutaneous and open techniques.

Together, these findings contribute to refining treatment protocols by promoting individualized surgical strategies informed by patient condition, injury patterns, and technological advancements. The integration of AI-driven models, advanced imaging, and minimally invasive techniques further supports evidence-based, precision-driven care. Moving forward, these insights should guide future clinical trials and the development of standardized treatment algorithms to enhance patient outcomes in pelvic trauma management.

## Data Availability

All data used is listed in references.

## Abbreviations

The following abbreviations are used in this manuscript:

DCO	Damage control orthopedics
ETC	Early total care
SIRS	Systemic inflammatory response syndrome
MODS	Multiple-organ dysfunction syndrome
ICU	Intensive care unit
CT	Computed tomography
4D	Four-dimensional (imaging)
AI	Artificial intelligence
CRPIF	Closed reduction and percutaneous internal fixation
ORIF	Open reduction and internal Fixation
DVT	Deep vein thrombosis
PE	Pulmonary embolism
VTE	Venous thromboembolism
AR	Augmented reality
AUC	Area under the curve
LC1b	Lateral compression Tybe 1b (Young–Burgess classification)
INFIX	Internal fixator
